# Knowledge gaps and acceptability of abbreviated alcohol screening in general practice: a cross-sectional survey of hazardous and non-hazardous drinkers

**DOI:** 10.1186/s12875-015-0290-1

**Published:** 2015-06-20

**Authors:** Alexander Isted, Francesco Fiorini, Taavi Tillmann

**Affiliations:** King’s College London, School of Medicine, London, UK; Department of Epidemiology & Public Health, University College London, London, UK

**Keywords:** Alcohol drinking, Binge drinking, General practice, Primary health care, Early medical intervention, Health education, Mass screening

## Abstract

**Background:**

General practice provides a unique setting where hazardous alcohol consumption can be screened for and behavioural interventions can be implemented in a continuous care model. Our aim was to assess in a general practice population, the prevalence of hazardous drinking, the knowledge and attitudes surrounding alcohol, and the acceptability of brief interventions in alcohol.

**Methods:**

A cross-sectional survey in a practice in South London, performed as part of a wider service evaluation. Questionnaires were offered to adult patients awaiting their appointments. Responses were stratified according to hazardous drinking, as per the abbreviated ‘Alcohol Use Disorders Identification Test’ (AUDIT-C).

**Results:**

Of 179 respondents (30 % male), 34 % yielded an AUDIT-C ≥5 and 18 % reported that they never drink alcohol. Male and Caucasian patients were more likely to self-report hazardous drinking, who in turn were more likely to believe in the health benefits of moderate consumption. Little over half of patents thought that alcohol is a risk factor for cancer and were misinformed of its calorific content, suggesting two targets for future improvement. Patients’ knowledge about what is a single ‘unit’ of alcohol was below that expected by random chance 66 % agreed that alcohol screening should feature in all GP consultations.

**Conclusions:**

While awareness of alcohol related health risks is generally good, future efforts may benefit from focusing on the association with cancer and calories. Our findings question the utility of the ‘unit’ system, as well as dissemination of suggested ‘health benefits’ of moderate consumption. General practice initiatives in screening and brief advice for alcohol deserve further study.

## Background

Alcohol consumption is a global problem with extensive chronic health implications [1]. Globally, it is attributable to 5.9 % of all deaths (3 million each year) [2]. Moreover, the situation has worsened: in 1990, alcohol was the 8th most important cause of death and disability in the world, but by 2010 it had risen to 5th, outranking BMI, physical inactivity, high cholesterol and not breastfeeding [3]. In the UK, alcohol misuse is prevalent, with hazardous drinking rates of 16 % for women and 33 % for men aged 16 years and over [4], and with dependence affecting 4 % of women and 9 % of men aged 18–64 [5]. Estimated societal costs exceed £20 billion per annum, through costs to the National Health Service (NHS), crime and lost productivity [6] – costs equal to approximately a fifth of the entire NHS budget.

Current UK Department of Health guidelines recommend against regularly exceeding 3–4 units per day for men and 2–3 units per day for women [7]. The Royal College of Physicians recommends a maximum of 21 units per week for men and 14 units per week for women, with 2–3 alcohol-free days per week [8]. Despite the majority of adults in the UK having heard of measuring alcohol consumption in units, fewer are aware of the maximum recommended intake [9] and fewer still understand what a unit accurately translates to in terms of drinks [10]. While strategies to inform the public of the harms of tobacco use have been moderately successful [11], misconceptions surrounding the risks of excessive alcohol intake remain prevalent [12–15]. Much of this existing literature draws from large population-based surveys, but there is limited literature into whether a similar set of knowledge gaps exists in the demographic of patients visiting their general practitioners. It is important to explore these knowledge gaps in primary care populations, since this is a unique ‘front line’ where hazardous alcohol consumption can be screened for, and behaviour change initiatives can be implemented, with long-term support and continuity of care. It has recently been shown that screening for hazardous drinking in general practice with implementation of a brief intervention can prompt patients to reduce their consumption by 4–5 units per week, a change that is sustained by 12-months of follow-up [16, 17]. Measures that have shown benefit include simple provision of an information leaflet, brief lifestyle advice during a consultation, or more lengthy and detailed counseling sessions [18]. The merits and technical feasibility of universal screening and brief intervention in general practice have been widely explored [18, 19] and are not the primary focus of this study. However, should such approaches be implemented, their success would be predicated on patient acceptability, which has received little attention in the literature. Furthermore, more detailed understanding of the knowledge and attitudes of at-risk drinkers could allow such approaches to be tailored and in doing so potentially increase their effectiveness.

The aim of this study was to sample patients at a typical UK general practice to determine: 1) patients’ *knowledge* and *beliefs* surrounding hazardous alcohol consumption and its risks to health; 2) the relationship between patients’ alcohol consumption patterns and their knowledge and beliefs of alcohol; and 3) patients’ acceptability of general practice based alcohol screening.

## Methods

### Participants & setting

Anonymous written questionnaires were offered in person by the first two authors to a population of general practice patients at Torridon Road Medical Practice in South East London, as they waited for their consultation between November 2014 and January 2015. This offer was made in conjunction with a broader offer to evaluate the quality of their service in routine fashion. This practice reported a 2012 Index of Multiple Deprivation (IMD) score of 24.3 placing it in the fifth decile nationally, representing a socioeconomic demographic comparable with the UK average. Health parameters for patients registered at the practice, including: the proportion of patients with a long-standing health condition (53.5 %); estimated smoking prevalence (17.9 %); obesity rates (9.3 %); and the proportion of patients reporting a mental health problem (2.6 %) were not significantly different from the rest of the UK [20]. All patients seated in the waiting area at any one time were approached to limit selection bias. Patients were eligible if aged 18 or over, and able to communicate and read in English. Patients read the brief cover letter explaining the purpose of the questionnaire and subsequently gave informed, verbal consent.

### Questionnaire

The questionnaire was developed to gather data under four key domains: patient demographics; level of alcohol consumption; knowledge; and beliefs. Questions A-D provided demographic information (age, sex, ethnicity and highest level of education). Questions E-G were used to quantify alcohol consumption by applying the ‘Alcohol Use Disorders Identification Test’ (AUDIT-C) three-item alcohol screen. Patients selected the answer that most closely matched the frequency that they had a drink containing alcohol and how often they had consumed 6 or more drinks on a single occasion in the previous year. They were asked to quantify the number of each of a selection of common alcoholic drinks that they consumed on a typical day of drinking. The AUDIT-C is a well-validated, abbreviated test for hazardous alcohol consumption and risk of alcohol dependence with a previously described scoring system from 0–16 [21]. A cut-off score of 5 or more was selected as it consistently provides strong sensitivity and specificity for men and women of ranging demographics (area under the receiver operator characteristic for hazardous drinking: 0.984 for men and 0.963 for women; and for dependence: 0.870 for men and 0.901 for women) [22]. Questions 1–15 addressed a number of knowledge-based questions, including awareness and understanding of the ‘unit’ system and the potential health harms of alcohol – both to the individual physiologically and on a wider epidemiological scale. Correct answers were agreed on by the authors and were confirmed by independent experts (see acknowledgements). Questions 16–19 were used to explore patient beliefs surrounding alcohol use in the broader context of society, how it currently stands, and whether this could be addressed in the setting of general practice. The belief- and knowledge-based questions were of a multiple-choice or true/false format. Patients indicated their selection by ticking one response that best matched their personal understanding.

### Analysis

Responses to each question were summarised as per cent ‘positive’ or per cent ‘correct’ where appropriate. Respondents were stratified in accordance with AUDIT-C scores (AUDIT-C score ≥5 denoting ‘hazardous drinking and risk of alcohol dependence’ and <5 denoting ‘non-hazardous drinking’) and group differences were assessed for statistical significance using *χ*^2^ and Mann Whitney U tests for comparing frequencies and continuous variables respectively. All analyses were performed using SPSS statistics package (IBM, Version 22.0). P values <0.05 were regarded as significant.

## Results

Of the 200 patients who agreed to participate in the study, 21 were excluded due to suboptimal completion of their questionnaire (<75 % completion), leaving 179 respondents whose data were analysed. Patient age ranged from 18–88 years (median 46 years) and did not differ significantly from that of adult patients registered at the practice. The sample population included fewer male and Caucasian patients than the reported practice population [20] (30.3 % versus 46.5 % male (*p* < 0.01), and 40.1 % versus 68.0 % Caucasian (*p* < 0.01)). There were no significant differences between other ethnicities, meaning that most of the minority ethnic groups were slightly overrepresented in our sample. In comparison with the 2011 borough census data [23], our sample had slightly lower maximal educational qualifications (a greater proportion with no formal qualifications (26.2 % versus 17.7 % (*p* < 0.01)); less likely to have GCSEs or equivalent (General Certificate of Secondary Education, typically attained in the UK at age 16) (14.9 % versus 23.6 % (*p* < 0.01)); less likely to have a degree level qualification (13.7 % versus 38.0 % (*p* < 0.01)). However, a greater proportion of people had completed their education to A levels, typically attained at age 18 (45.2 % versus 10.8 % (*p* < 0.01)), giving an overall mixed pattern of educational differences.

In total, 60 patients (33.5 %) reported patterns of alcohol drinking that yielded an AUDIT-C score ≥5, consistent with hazardous drinking and risk of alcohol dependence. A further 32 (17.9 %) patients reported that they never drink alcohol. When asked at what frequency patients consumed 6 or more drinks on a single occasion (a measure of binge drinking used in the AUDIT-C), 52.2 % selected ‘never’; 25.3 % ‘less than monthly’; 9.0 % ‘monthly’; 9.6 % 'weekly'; and 2.8 % ‘daily or nearly daily’. While debate remains about the best way to operationalise ‘binge drinking’, we used the criteria from the Health Survey for England and NHS recommendations (>8 units in single session for men, and >6 units for women [24, 25]). Of our high-risk patients (AUDIT-C ≥5), 55 % drank to a level consistent with ‘binge drinking’. Two patients who screened negative for the AUDIT-C (score <5) also qualified as ‘binge drinking’ but did so infrequently thereby not attaining sufficient points to screen AUDIT-C positive. Males were almost three times more likely to screen positive than females (62.3 % versus 21.8 % (*p* < 0.01)); and Caucasian patients were almost twice as likely to screen positive than other ethnicities (46.4 % versus 25.2 %, *p* < 0.01)). Age and level of education had no significant bearing on screening AUDIT-C positive.

Table 1 outlines the knowledge and belief-based questions, where applicable the correct answer, and the proportion of patients who made the correct or positive selection where appropriate. The knowledge-based questions most frequently answered incorrectly were: “alcohol is a stimulant” (61 % of respondents believed incorrectly that this was true); “a can of regular coke has more calories than a pint of beer” (51 % believed incorrectly that this was true); and “drinking more alcohol than recommended can cause cancer” (40 % believed incorrectly that this was false).

**Table 1:** Patient responses to knowledge and belief-based questions

	Question	Correct Response	Proportion of correct/positive responses			Sig. (*p*)
			All Patients	AUDIT-C <5 (n = 119)	AUDIT-C ≥5 (n = 60)	
1	Have you heard of the term ‘unit of alcohol’ before?	n/a	92.70 %	91.50 %	94.90 %	0.415
2	Which is closest to 1 unit of alcohol?	B	21.6 %	24.8 %	15.5 %	0.164
	**A)** Glass of wine;					
	**B)** Single measure of whisky;					
	**C)** Pint of beer;					
	**D)** Bottle (330 ml) of lager					
3	What’s the maximum recommended weekly alcohol intake for someone of your sex?	Males (B); Females (A)	71.4 %	77.5 %	59.6 %	0.015*
	**A)** 14 units					
	**B)** 21 Units;					
	**C)** 28 Units;					
	**D)**35 Units					
4	It is safer to drink your maximum weekly recommended amount of alcohol spread over more days rather than all in one day	T	83.8 %	82.9 %	85.0 %	0.722
5	A can of regular coke has more calories than a pint of beer	F	48.9 %	52.1 %	42.4 %	0.221
6	Alcohol improves sleep	F	84.7 %	88.0 %	78.3 %	0.089
7	Alcohol can be fattening	T	93.9 %	92.5 %	96.4 %	0.324
8	Alcohol can reduce fertility in:	D	76.4 %	74.6 %	80.0 %	0.420
	**A)** Men;					
	**B)** Women;					
	**C)** Neither men nor women;					
	**D)** Both men and women					
9	Alcohol is a stimulant	F	39.2 %	39.3 %	39.0 %	0.966
10	HIV kills more people worldwide than alcohol	F	86.0 %	86.1 %	86.0 %	0.983
11	Alcohol kills brain cells	T	92.7 %	94.1 %	90.0 %	0.361
12	In the UK, there are more people addicted to alcohol than to marijuana	T	94.9 %	93.2 %	98.3 %	0.141
13	Men are affected by alcohol to the same level as women of the same height and weight	F	72.9 %	70.1 %	78.3 %	0.243
14	Drinking a small amount of alcohol can be good for one’s health	n/a	65.5 %	55.6 %	85.0 %	<0.001*
15	Drinking more alcohol than recommended can cause:					
	**A)** Liver problems;	T	100.0 %	100.0 %	100.0 %	n/a
	**B)** Blindness;	F	65.1 %	63.6 %	67.9 %	0.590
	**C)** High blood pressure;	T	93.1 %	91.4 %	96.6 %	0.196
	**D)** Cancer	T	60.0 %	62.3 %	55.4 %	0.386
16	Do you think that alcohol is a significant problem in the UK?	n/a	94.4 %	94.9 %	93.3 %	0.665
17	Are alcohol-drinking health guidelines publicised well enough?	n/a	32.0 %	30.3 %	35.6 %	0.472
18	Should questions on alcohol habits feature in every GP consultation?	n/a	66.1 %	69.5 %	59.3 %	0.178
19	You would know how to find more information, advice and help regarding alcohol use:					
	**A)** Strongly disagree	n/a	5.2 %	4.3 %	6.8 %	0.493
	**B)** Disagree	n/a	7.5 %	8.7 %	5.1 %	0.391
	**C)** Neither agree nor disagree	n/a	21.8 %	23.5 %	18.6 %	0.465
	**D)** Agree	n/a	48.3 %	47.8 %	49.2 %	0.868
	**E)** Strongly agree	n/a	17.2 %	15.7 %	20.3 %	0.439

*Knowledge and belief-based questionnaire response data for all patients; patients with an AUDIT-C score of* <5; and *patients with an AUDIT-C score of ≥5, denoting ‘hazardous drinking and risk of alcohol dependence’. Where appropriate, the correct response is given. χ* ^2^ tests were used to compare frequencies between the AUDIT-C negative and AUDIT-C positive groups. * denotes a P-value <0.05 and is considered significant

## Discussion

The study highlights how in a typical UK General Practice setting, patients’ knowledge is often lacking in understanding the ‘unit’ system’, risks of cancer, the calorific content of alcoholic drinks and possible health benefits of moderate consumption. It also provides interesting patient insight into alcohol management in primary care, particularly the positive patient attitudes towards future alcohol screening in GP consultations.

In a large study commissioned by the European Union, consumption of 6 of more drinks on a single occasion in the UK was reported as: never (31 %); less than monthly (20 %); monthly (14 %); weekly (20 %); and daily or nearly daily (14 %) [26]. Within this study’s patient sample, the corresponding values were 52 %, 25 %, 9 %, 10 % and 3 %. This lesser proportion of patients who reported binge drinking may reflect the fact that the most at-risk drinkers may be unlikely to present to general practice, underreporting from social desirability bias, and/or lower prevalence of binge-type pattern of drinking among non-Caucasian ethnic groups and females who were overrepresented in our sample. Despite this, 33.5 % of patients exceeded the AUDIT-C threshold, qualifying as ‘hazardous drinking with risk of alcohol dependence’. Of these, only 55.0 % met criteria for ‘binge drinking’. This demonstrates one limitation of AUDIT-C as it doesn’t effectively discriminate between those who occasionally drink to great excess, from those who chronically abuse in smaller quantities. The former is more at risk of accidents, injuries and possibly arrhythmias while the latter more from liver and neoplastic disease [27]. At present, tools like the AUDIT-C cannot differentiate between these subtypes of hazardous drinking. Being male and Caucasian were both factors significantly associated with higher intake, concurring with the wider literature [23, 28].

Comprehensive appreciation of alcohol consumption guidelines relies on understanding of all three components of the unit system: (i) what a unit is, (ii) how many units one can safely consume and (iii) what a unit equates to in terms of drinks. Awareness of the term “unit of alcohol” was highly prevalent at 93 %. However, only 71 % of respondents were able to correctly identify the maximum recommended intake. When stratified by AUDIT-C score, the proportion of patients who correctly identified their value was significantly lower in the higher intake group (59.6 % versus 77.5 %, *p* = 0.02). Fewer still were able to correctly select “a single measure of whisky” as the drink closest to one unit of alcohol (21.6 % overall, 15.5 % in the higher intake group verses 24.8 % in the lower intake group (*p* = 0.16)). This suggests that attempts to educate the public about safe alcohol unit levels are failing disproportionately to reach those at most risk. Moreover, given that the multiple-choice format of the questionnaire incurs a 25 % chance of selecting the correct answer by probability alone, the lack of awareness across the whole sample is particularly marked. This knowledge gap is not limited to the general practice population. The charity Drinkaware, despite being funded by the alcohol industry, has also accepted that 70-85 % of 18-24-year-olds cannot identify the unit content of lager and wine respectively [10]. Our findings reiterate previously voiced limitations of efforts to educate the population about the unit system [9, 10].

Misconceptions surrounding the mechanisms and harms of alcohol are well established in the literature. Alcohol, a known carcinogen, is attributed to 4 % of all cancers in the US, directly associated with liver, oral and oesophageal cancers and is a significant risk factor for colorectal and breast cancers [29]. Sanderson *et al.* asked participants to list lifestyle-based risk factors for cancer, with only 14 % of respondents selecting “excessive alcohol consumption” [12]. A large Irish survey revealed that only 49 % of respondents thought alcohol could increase risk of breast cancer and only 65 % knew of the increased risk of bowel cancer [13]. In a 2012 survey of Southern Australians, Bowden *et al.* found that only 37 % of respondents considered alcohol to be a ‘very’ or ‘extremely’ important risk factor for cancer, in comparison with 72 % for pollution and 92 % for smoking cigarettes [30]. These themes are mirrored in our sample. While all respondents correctly stated that excess alcohol was associated with “liver disease”, and the majority associated it with hypertension (93 %), only 60 % thought there was a link to cancer. A greater proportion considered there to be a link with blindness (65 %), which could be considered a risk secondary to increased incidence of hypertension, but is not thought to be a direct risk with legal alcohol products. The vast majority (93 %) agreed that alcohol “kills brain cells”, a point of contention in the literature with some evidence of chronic alcohol abuse causing a degree of neuronal loss [31]. Its inclusion in the questionnaire was not to debate the scientific merits of the statement itself but to highlight the relative emphasis of different potential risks held by patients. Awareness of the risk of cancer, a highly prevalent and incontestable complication of alcohol excess, was far less than perhaps less tangible and more theoretical risks, amongst patients. Among the general population, cancer is highly feared, and given more importance than other diseases. This can be seen in the public outcries that led to the establishment of the Cancer Drugs Fund [32]. Increased efforts to raise public awareness about the link between cancer and alcohol may yield stronger motivation to cut back on drinking when compared to other, less feared or less well understood diseases, such as hypertension.

The majority of respondents considered alcohol to be “fattening” but fewer than half thought that a pint of beer contained more calories than a can of regular coke (approximately 180 versus 139 kcal). This suggests an underestimation of the true calorie content of alcoholic drinks, reflected in a survey conducted by the charity Alcohol Concern, which reported that 82 % of respondents were unable to correctly identify the calorific content of a standard pint of beer or a standard glass of wine [33]. Improving patient education regarding the calorie content of alcoholic beverages may provide individuals with added impetus to control intake, particularly for those who are conscious of body image, for whom less visible consequences like hypertension may be ignored. The literature on this is scarce and research is needed to gauge the efficacy of patient education interventions specifically focussing on alcohol’s calorie content. There is, however, mounting evidence that calorie-labelling of alcoholic beverages is an effective intervention, which the public is in favour of implementing [34, 35].

Studies purporting that “drinking a small amount of alcohol can be good for one’s health” have been widely publicised in mainstream media, and consequently the belief is prevalent in the sample population (66 %). Observational evidence has suggested that low-level alcohol consumption (strictly below the UK’s maximum recommended intake) may have a modest protective effect against coronary heart disease, an effect lost and reversed with excessive consumption [36, 37]. However, these observational studies may be confounded by a variety of biases, which novel Mendelian randomisation studies have sought to remove. More recent evidence suggests that low levels of alcohol may not hold any protective effect at all [38]. Nonetheless, despite this scientific debate, it was interesting to see that our hazardous alcohol drinkers were more likely to believe in the health benefits of low levels of alcohol consumption when compared to the non-hazardous drinkers (85.0 % versus 55.9 %, *p* < 0.01). This question accounted for the greatest divide in responses between the two groups. Either information about the health-benefits of moderate alcohol consumption is perversely being targeted at high-risk drinkers, or high-risk drinkers choose to retain this information better as a possible method of overcoming cognitive dissonance (the inner conflict between knowing that high-risk drinking is harmful and wanting to cut down, yet continuing to drink in a high-risk manner). This echoes the widely held notion that publicising the health benefits of moderate consumption to the general population is likely to lead to substantial overall harm, and should be avoided [39].

Patients were aware that alcohol is responsible for greater mortality than HIV and more cases of dependence than marijuana, tying in with the fact that in the broader context of alcohol’s impact on society, patients almost universally (94 %) considered it a ‘significant problem in the UK’. In contrast, only a minority of patients (32 %) believed national alcohol guidelines to be ‘publicised well enough’ and this is reflected in poor understanding of the unit system. Given that a growing body of evidence suggests that the ‘unit’ based system is failing more than 25 years since its introduction into UK guidelines [9, 10], exploration of simpler models may be needed.

Respondents were split in response to “should questions on alcohol habits feature in every GP consultation?” with the majority (66 %) agreeing overall. This mirrors the limited existing literature, predominantly conducted in the accident and emergency setting [40, 41] and warrants further investigation in primary care. When asked whether patients felt they “would know how to find more information, advice and help regarding alcohol use”, the majority (66 %) of responses were positive (‘agree’ and ‘strongly agree’) with 13 % responding negatively (‘disagree’ and ‘strongly disagree’). Level of consumption had no bearing on opinions. While encouraging, more can be done to inform patients about the role of the general practitioner in provision of education and support should they need it.

Strengths of the study lie in its moderate sample size and the use of the well-validated AUDIT-C screening test (albeit originally validated in the format of verbal administration rather than as part of a written questionnaire). Limitations principally lie in the nature of a written survey-based study with questionnaires issued opportunistically to patients in the practice’s waiting area. Sampling and response bias, and the fact that recording the number of patients who declined to participate was not part of the study design, may influence how accurately these findings represent those of the wider patient population. This was reflected in the reported demographics of respondents. There were significantly fewer males and Caucasians than the registered patient population, and a lower maximum attained level of educational compared to the borough as a whole.

Extrapolation of this single practice study’s findings should clearly be cautious, and results are principally being used in the context of service evaluation to guide healthcare promotion initiatives within the practice. However, this questionnaire provides a useful, practical and reproducible tool, which could be employed for further research to assess the validity of these findings in a range of primary care settings.

Proposed initiatives include more targeted patient education to tackle overlooked knowledge gaps and misconceptions, with a particular focus on the unit based system of alcohol measures, the calorie content of alcoholic drinks, and its most relevant potential risks with a greater emphasis on cancer. Furthermore, the high prevalence of problem drinking, coupled with patient acceptability, warrants continued discussion on the use of an abbreviated score as a screening tool in general practices. Rolling out AUDIT-C screening in general practice requires a comprehensive cost-effectiveness model and pilot evaluation, and our preliminary results suggest that such research may be warranted.

## Conclusions

While awareness of alcohol related health risks is generally good, future efforts may benefit from focusing on the association with cancer and calories. Our findings question the utility of the ‘unit’ system, as well as dissemination of suggested ‘health benefits’ of moderate consumption. General practice initiatives in screening and brief advice for alcohol deserve further study.

## Abbreviations

AUDIT-C: Alcohol Use Disorders Identification Test
GCSE: General Certificate of Secondary Education
GP: General practitioner
NHS: National Health Service
